# Protein inhibitor of retinal membrane guanylyl cyclase rescues photoreceptors from degeneration induced by mutated guanylyl cyclase activating protein

**DOI:** 10.1016/j.jbc.2026.113636

**Published:** 2026-10-14

**Authors:** Lyuqi Tan, Igor V. Peshenko, Elena V. Olshevskaya, Alexander M. Dizhoor

**Affiliations:** 1 Pennsylvania College of Optometry, Drexel University, Elkins Park, Pennsylvania USA; 2 Graduate School in Biomedical Sciences and Professional Studies, College of Medicine, Drexel University, Philadelphia, Pennsylvania USA; 3 Department of Neurobiology and Anatomy, College of Medicine, Drexel University, Philadelphia, Pennsylvania USA

**Keywords:** cyclic GMP, cGMP, eye, calcium binding proteins, GCAP, guanylate cyclase, guanylyl cyclase, *GUCY2D*, *GUCA1A*, photoreceptor, RetGC, retinal degeneration

## Abstract

Mutations in the *GUCA1A* gene coding for calcium-sensor protein GCAP1 cause congenital cone and cone–rod degenerations by disabling Ca^2+^-dependent reduction of cGMP synthesis in photoreceptors in the dark. Engineered protein inhibitor of guanylyl cyclase (PIGCY) effectively prevented *in vitro* stimulation of retinal membrane guanylyl cyclase (RetGC) by Y99C or E155G GCAP1, most commonly causing *GUCA1A* retinopathy. However, it was less effective against G86R and L176F GCAP1. PIGCY expressed in transgenic mouse rods (*PIGCY*^*Tg*^) did not reverse the abnormal Ca^2+^-sensitivity of RetGC in mice harboring Y99C GCAP1 (*Y99C*^*Tg*^) but reduced the overall cGMP production rate in dark-adapted *PIGCY*^*Tg*^*Y99C*^*Tg*^ retinas. PIGCY markedly slowed the progression of photoreceptor degeneration in *Y99C*^*Tg*^ mice: 84% of photoreceptor nuclei remained after 6 months in *PIGCY*^*Tg*^*Y99C*^*Tg*^ compared with only 30% in *Y99C*^*Tg*^ littermates. The histological layer of rod outer segments in the posterior retina was much better defined in aged *PIGCY*^*Tg*^*Y99C*^*Tg*^ mice than in *Y99C*^*Tg*^ littermates. Electroretinography (ERG) showed that PIGCY also dramatically rescued the light sensitivity of the dark-adapted *Y99C*^*Tg*^ rods. Whereas scotopic b-wave amplitude (rod ERG) diminished sixfold in *Y99C*^*Tg*^ compared with the wild type, it remained 80% of normal amplitude in *PIGCY*^*Tg*^*Y99C*^*Tg*^ littermates. ERG a-wave amplitude, generated by hyperpolarizing photoreceptors in response to a bright flash, was reduced more than tenfold in *Y99C*^*Tg*^ but only by 35% in *PIGCY*^*Tg*^*Y99C*^*Tg*^. The results indicate that PIGCY can be considered for prospective gene therapy for some GCAP1 mutations causing dominant *GUCA1A* retinopathy.

Changes in cGMP concentrations in rod and cone outer segments regulate the permeability of the cyclic-nucleotide gated (CNG) channels in the photoreceptor plasma membrane, thereby mediating both excitation and recovery of the light response (reviewed in 1, 2, 3, 4). Two opposing processes maintain the cGMP flux in the outer segment. During excitation, the light-activated enzymatic cascade, rhodopsin–transducin–phosphodiesterase 6 (PDE6), activates cGMP hydrolysis (reviewed in 1, 2). During recovery, cGMP synthesis by retinal membrane guanylyl cyclase (RetGC) (reviewed in 3, 4, 5) accelerates CNG channel opening to help restore photoreceptor light sensitivity and enable their adaptation to background light. Negative calcium feedback on RetGC mediated by guanylyl cyclase–activating proteins (GCAPs) controls RetGC activity between light- and dark-adapted states of photoreceptor. GCAPs accelerate RetGC after illumination, when Ca^2+^ influx through the CNG channels stops and GCAPs are converted to their Mg^2+^-liganded, “RetGC-activator,” state. When Ca^2+^ influx resumes, Ca^2+^GCAPs decelerate RetGC activity and maintain it at low levels in the dark 3, 6. However, multiple mutations in the *GUCA1A* gene coding for GCAP1 can alter the normal Ca^2+^ feedback, thus making the RetGC1:GCAP1 complex less sensitive to deceleration by calcium in the dark 7, 8, 9, 10. Such *GUCA1A* mutations cause severe forms of congenital blindness, dominant cone and cone–rod degenerations. In mouse models harboring GCAP1 variants causing dominant human *GUCA1A* retinopathies, increases in cGMP and free Ca^2+^ concentrations caused by deregulated cGMP synthesis in the dark trigger photoreceptor death 11, 12. The progression of photoreceptor degeneration can be slowed by artificially increasing cGMP hydrolysis in the dark 12, 13, either by mutant rhodopsin activating endogenous PDE6 (14) or by supplying the affected photoreceptors with constitutively active recombinant cGMP phosphodiesterase 5 (PDE5) (13).

In the present study, we also explored an alternative strategy for counteracting the molecular mechanism of dominant *GUCA1A* retinopathy by using an engineered protein inhibitor of guanylyl cyclase (PIGCY) (15). PIGCY was developed by converting GCAP1 into a form that retains its high specificity and affinity for RetGC1 but cannot activate RetGC (15). Consequently, PIGCY uncouples RetGC from endogenous GCAP1 and thus prevents its abnormal activity in the dark caused by deregulation of the negative Ca^2+^-feedback 15, 16. This approach was successfully tested in a mouse model harboring a mutant form of RetGC1 isozyme, which causes human dominant *GUCY2D* retinopathy (16) resulting from deregulation of the negative Ca^2+^ feedback on RetGC1 3, 4, 7, 8, 9, 10. We reasoned that PIGCY should not only displace normal GCAP1 from a mutated RetGC1, but also displace mutant GCAP1 from the complex with the normal endogenous RetGC1. We demonstrate in this study that PIGCY expression in a mouse model slows retinal degeneration caused by dominant GCAP1 mutations and preserves light sensitivity in the rescued retina.

## Results

### Differential effects of PIGCY on inhibition of RetGC1 in complex with disease-causing GCAP1 mutants

To assess sensitivity of different retinopathy-causing GCAP1 variants to PIGCY-mediated inhibition, PIGCY was tested *in vitro* by using recombinant RetGC1 (also referred to as GC-E, or GUCY2D) 17, 18, 19, the main RetGC isozyme in photoreceptors 20, 21, activated by several such GCAP1 mutants: Y99C 6, 7, E155G (22), G86R (23), and L176F (24). The Tyr^99^ substitution with Cys most frequently causes dominant retinopathy among multiple *GUCA1A* mutations found in various ethnic populations 9, 10, 25, whereas E155G, G86R, and L176F variants, also causing progressive retinal degeneration, are among less frequently reported (25). All tested GCAP1 mutants equally strongly shifted Ca^2+^-sensitivity of RetGC1 to a much higher Ca^2+^ range than wild-type GCAP1 (Fig. 1).

**Figure 1:**
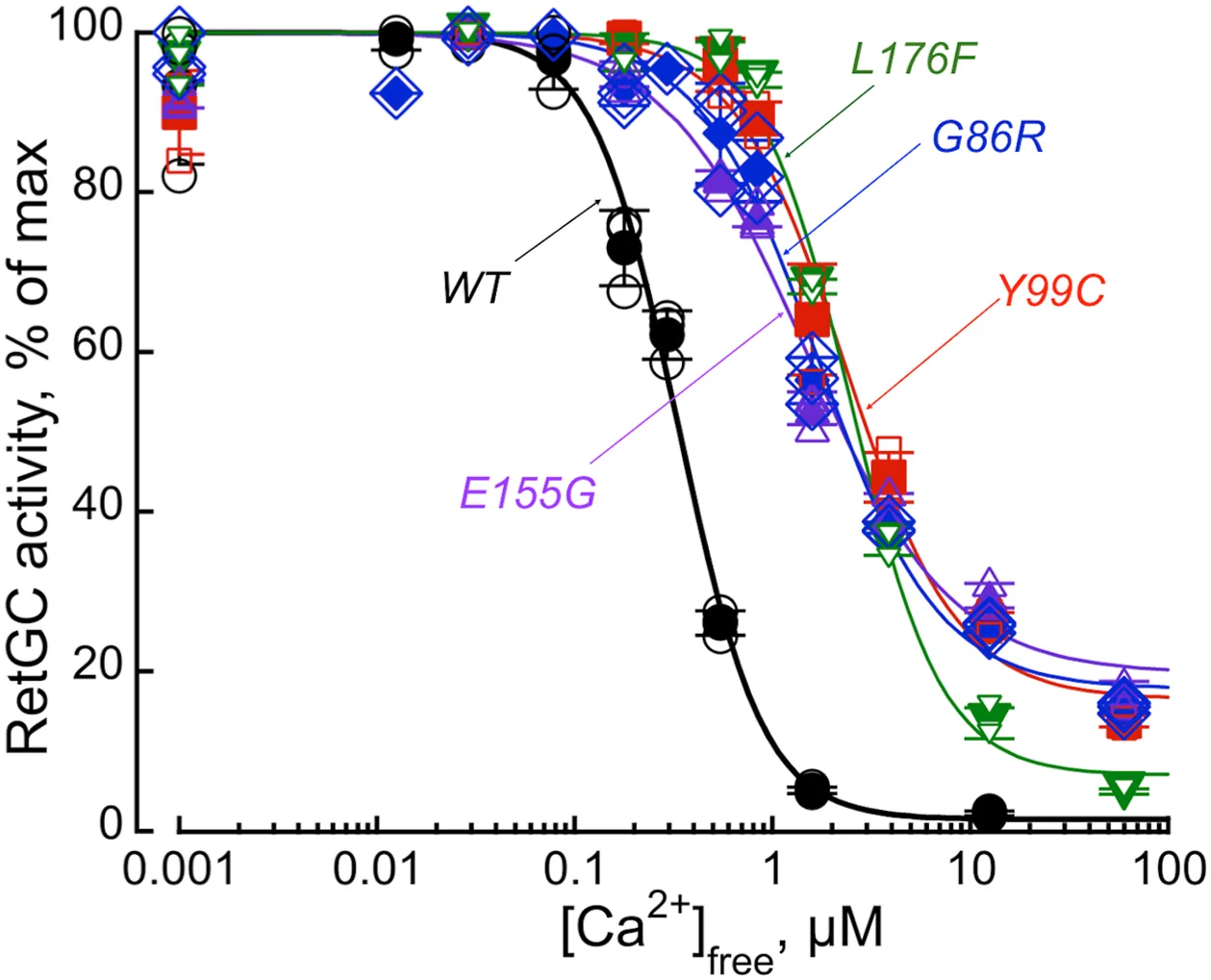
**Calcium-sensitivity of RetGC1 regulation by GCAP1 mutants causing dominant *GUCA1A* retinopathies.** HEK 293 cells membrane fraction containing recombinant human RetGC1 reconstituted with 10 μM WT (○, •), E155G (△, ▲), G86R (◇, ◆), L176F (▽, ▼) or Y99C (□, ▪) GCAP1 was assayed in the presence of 2 mM Ca^2+^/EGTA/Mg^2+^ buffer at indicated Ca^2+^ concentrations and 0.9 mM Mg^2+^. The RetGC activities were normalized per maximal activity for each GCAP1 mutant, and the data was fitted assuming a sigmoidal function, *a = (100 − a*_*min*_*)/(1+([Ca]/[Ca] _half_)*^*h*^*) + a*_*min*_, where *a*_*min*_ is the fitted minimal RetGC1 activity at high free Ca^2+^ concentrations, *[Ca]* is the free Ca^2+^ concentration, *[Ca]_half_* is the concentration of Ca^2+^ required for half-maximal inhibition, and *h* is the Hill coefficient. Open symbols: independent replicates; closed symbols with error bars: mean ± SD. See other details in Experimental procedures.

Despite the similar change in Ca^2+^ sensitivity, we observed a substantial difference between the mutants in sensitivity of their RetGC1:GCAP1 complexes to PIGCY-mediated inhibition (Fig. 2). RetGC1 activation by 1 μM E155G or Y99C Mg^2+^GCAP1 was reduced in half at 74 nM and 112 nM PIGCY, respectively, with a modest increase in IC_50_ compared with wild-type GCAP1 (48 nM). However, both G86R and L176F Mg^2+^GCAP1 imparted much higher resistance to RetGC1 inhibition by PIGCY (IC_50_ 325 nM and 681 nM, respectively). Notably, these two mutations have been found to not only shift the Ca^2+^-sensitivity of the cyclase regulation but also dramatically elevate the affinity of GCAP1 for RetGC 23, 26. Because the Y99C GCAP1 mutant, the most frequent among those causing *GUCA1A* retinopathy (Human Gene Mutation Database (25)), displayed only slightly reduced sensitivity to PIGCY-mediated inhibition *in vitro* (Fig. 2), the effect of PIGCY on rod physiology and survival was further investigated using a mouse model harboring Y99C GCAP1 expressed under control of the rod opsin promoter (11).

**Figure 2:**
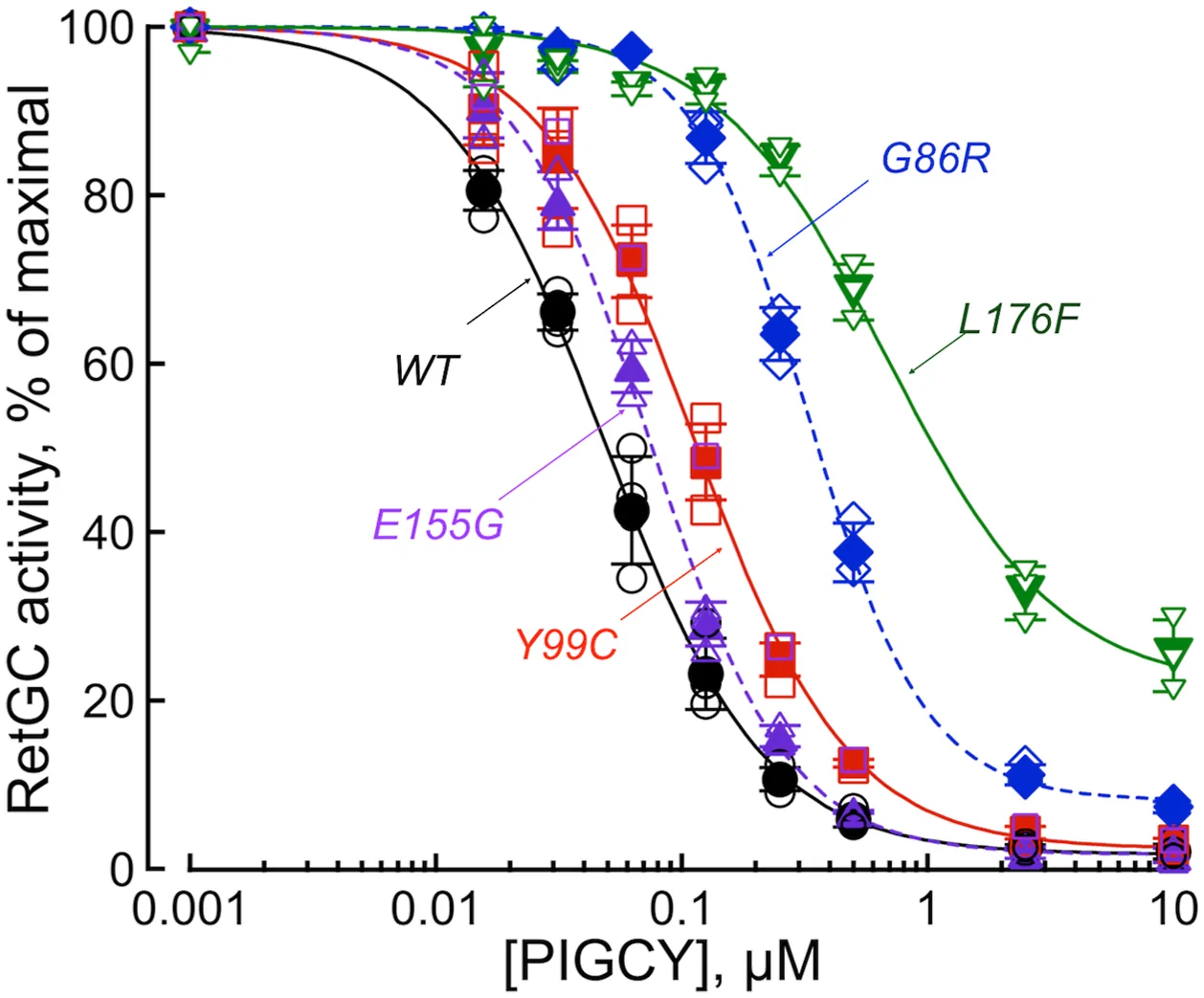
**Sensitivity to inhibition by PIGCY of RetGC1 activated by different GCAP1 mutants.** RetGC1 activated by 1.0 μM WT (○, •), E155G (△, ▲), G86R (◇, ◆), L176F (▽, ▼) or Y99C (□, ▪) GCAP1 at 2 mM EGTA and 10 mM total Mg^2+^ was assayed in the presence of the indicated concentrations of PIGCY; open symbols–independent measurements; filled symbols with error bars–mean ± SD; the data were fitted assuming the function *a* = (*100*−*a*_*min*_)/(*1* + (*[PIGCY]*/*IC*_*50*_)^*h*^)+*a*_*min*_, where *a* is RetGC1 activity, *a*_*min*_ is the fitted minimal residual RetGC1 activity at high PIGCY concentrations, *IC*_*50*_ is the PIGCY concentration causing half-maximal inhibition, and *h* is the Hill coefficient.

### PIGCY inhibits cGMP synthesis in transgenic retinas harboring Y99C GCAP1

*PIGCY*^*Tg*^*Y99C*^*Tg*^ hybrids were produced by cross-breeding of previously characterized *PIGCY*^*Tg*^ 15, 16 and *Y99C*^*Tg*^ (11) mouse models. As one of the most typical *GUCA1A* mutations causing dominant retinopathies, Y99C GCAP1 7, 8 provokes photoreceptor death by reducing the calcium sensitivity of cGMP synthesis, shifting outside the normal physiological range of free intracellular Ca^2+^ 11, 12. Transgenic expression of PIGCY in *PIGCY*^*Tg*^*Y99C*^*Tg*^ hybrid rods did not reverse the altered Ca^2+^ sensitivity of cGMP synthesis in the retina back to the normal range (Fig. 3*A*). Ca^2+^ concentration decelerating RetGC in half was two-fold higher than normal, 151 ± 44 nM (mean ± SD) *versus* 71 ± 15 nM in the wild type (unpaired *t* test *p* = 0.011) and remained in *PIGCY*^*Tg*^*Y99C*^*Tg*^ hybrid virtually identical to previously reported for *Y99C*^*Tg*^ (11) used for producing the hybrid (Fig. 3*A*).

**Figure 3:**
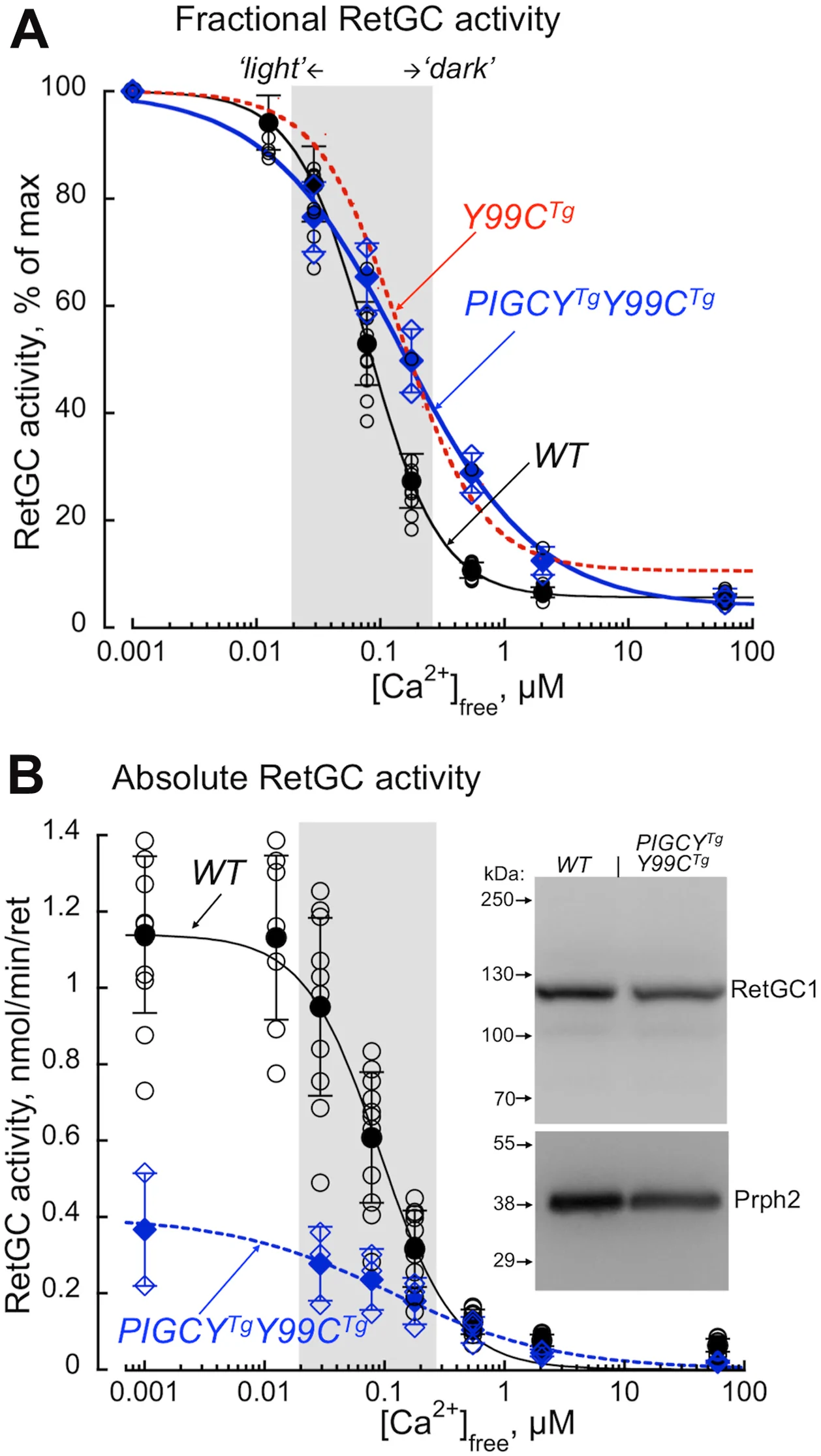
**PIGCY reduces RetGC activity deregulated by Y99C GCAP1 in mouse rods.** RetGC activity was assayed at variable free Ca^2+^ concentrations in the presence of 0.9 mM free Mg^2+^ in dark adapted wild type (○, •) and *PIGCY*^*Tg*^*Y99C*^*Tg*^ (◇, ◆) retinas aged 1 month. *A*, Ca^2+^-sensitivity of RetGC activity, normalized by the maximal activity in each independently assayed retina sample; *red dashed curve* represents Ca^2+^ sensitivity of the cyclase (*[Ca]half* ∼143 nM) in *Y99C*^*Tg*^ mouse line used for producing the hybrid (data from the original publication by Olshevskaya *et al.*, 2004 (11)). *B*, Actual cGMP production rates measured in the retina. Open symbols–independent retinal preparations; filled symbols with error bars–mean ± SD, fitted assuming a sigmoidal Hill function; *shaded area* approximates the range of free Ca^2+^ concentrations in mouse rod outer segments between light-adapted and dark-adapted states (27); *inset*–immunoblotting of proteins extracted from wild-type and *PIGCY*^*Tg*^*Y99C*^*Tg*^ retinas probed with anti-RetGC1 (*upper panel*) and anti-peripherin-2 antibody (*lower panel*).

PIGCY and Y99C GCAP1, who both have slightly lower than endogenous GCAP1 electrophoretic mobility (Fig. S1), are expressed in the parental *PIGCY*^*Tg*^ (15) and *Y99C*^*Tg*^ (11) mouse models at levels comparable to the endogenous wild-type GCAP1. Effect of PIGCY on expression of Y99C GCAP1 in *PIGCY*^*Tg*^*Y99C*^*Tg*^ hybrid could not be directly evaluated, because they have identical electrophoretic mobility (Fig. S1) and react with the same anti-GCAP1 antibody 11, 15. However, the still abnormal Ca^2+^ sensitivity of RetGC (Fig. 3*A*) indicated that PIGCY did not prevent expression of Y99C GCAP1.

Importantly, the Ca^2+^-sensitivity remaining shifted in *PIGCY*^*Tg*^*Y99C*^*Tg*^ did not entail an increase in the actual rate of cGMP production above normal at Ca^2+^ concentrations typical for dark-adapted rods (Fig. 3*B*), because PIGCY reduced the overall rate of RetGC activity in *PIGCY*^*Tg*^*Y99C*^*Tg*^ within the entire estimated range of free Ca^2+^ between light- and dark-adapted states of mouse rods (27). Below 20 nM Ca^2+^, the optimal conditions for RetGC activation by Mg^2+^GCAPs in the light 3, 4, 28, 29, 30, the rate of cGMP production in dark-adapted *PIGCY*^*Tg*^*Y99C*^*Tg*^ retinas decreased threefold, to 0.37 ± 0.14 nmol cGMP min^−1^ retina^−1^, from 1.14 ± 0.21 nmol cGMP min^−1^ retina^−1^ in wild type (unpaired *t* test *p* = 0.001). Even at 178 nM free Ca^2+^, concentration approaching the levels estimated for mouse rods in the dark 11, 27, RetGC activity in *PIGCY*^*Tg*^*Y99C*^*Tg*^, despite its lower sensitivity to deceleration by Ca^2+^ (Fig. 3*A*), remained below wild type: 0.18 ± 0.06 nmol cGMP min^−1^ retina^−1^ *versus* 0.32 ± 0.1 nmol cGMP min^−1^ retina^−1^ , respectively (*p* = 0.029) (Fig. 3*B*). As previously described for *PIGCY*^*Tg*^ (15), the dramatic reduction of RetGC activity in *PIGCY*^*Tg*^*Y99C*^*Tg*^ hybrid retinas was the result of its inhibition rather than loss of RetGC expression (Fig. 3*B*, inset).

### PIGCY preserves Y99C^Tg^ rods from rapid degeneration

The reduced RetGC activity prevented rapid loss of the photoreceptor layer in PIGCY^Tg^*Y99C*^*Tg*^ (Fig. 4). The outer nuclear layer comprised predominantly of rod nuclei (97% versus 3% of cone nuclei (31)) remained thicker than in *Y99C*^*Tg*^ littermates at different ages (Fig. 4*A*). While the anterior retina looked similar in both *PIGCY*^*Tg*^*Y99C*^*Tg*^ and *Y99C*^*Tg*^ mice even after 8 months, the histological layers of their posterior retinas looked strikingly different at that age. *PIGCY*^*Tg*^*Y99C*^*Tg*^ posterior retinas retained all histological layers typical of normal retinas, where the layers formed by rod inner and outer segments were clearly identifiable. By contrast, *Y99C*^*Tg*^ littermates, along with the heavily compromised outer nuclear layer, had no clearly discernible rod outer segment layer (Fig. 4*B*).

**Figure 4:**
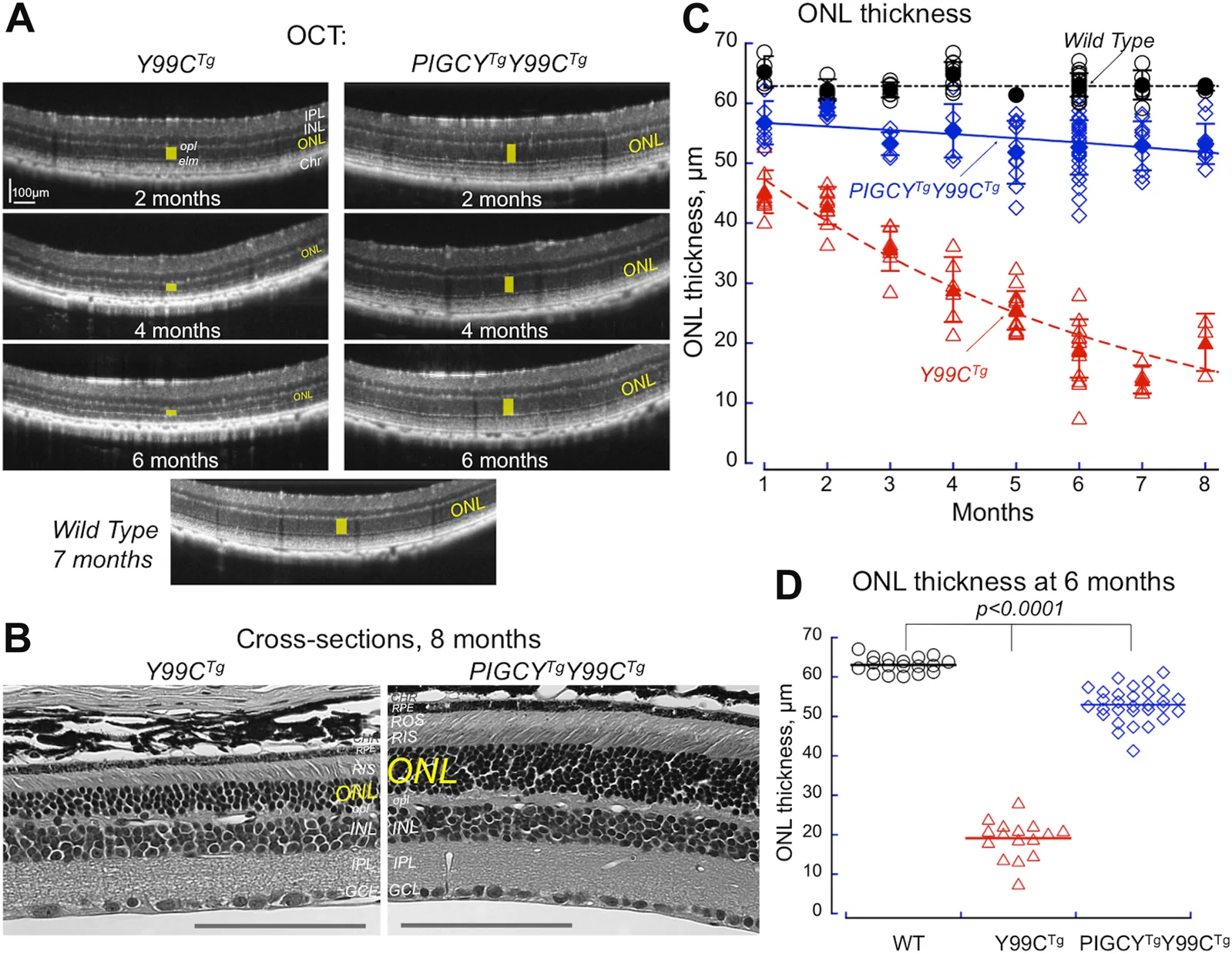
**PIGCY rescues *Y99C*^*Tg*^ rods from rapid degeneration.***A*, *B*, PIGCY preserves *Y99C*^*Tg*^ retinal structure; bars–100 μm. *A*, Representative retinal OCT images from *Y99C*^*Tg*^ (*left*), and *PIGCY*^*Tg*^*Y99C*^*Tg*^ (*right*) mouse eyes at different ages. The thickness of the outer nuclear layer (ONL) is marked yellow between *elm*–external limiting membrane, and *opl*–outer plexiform layer. *B*, Representative hematoxylin/eosin-stained aldehyde-fixed retinal cross-sections of *Y99C*^*Tg*^ (*left*), and *PIGCY*^*Tg*^*Y99C*^*Tg*^ (*right*) mouse retinas aged 8 months; *CHR*–choroid; *GCL*–retinal ganglion cell layer; *IPL*–inner plexiform layer, *INL*–inner nuclear layer; *IS*–rod inner segment layer; *ONL*–outer nuclear layer; *OPL*–outer plexiform layer; *ROS*–rod outer segment layer; *RPE*–retinal pigment epithelium. *C*, the ONL thickness measured by OCT in wild-type (•,○), *Y99C*^*Tg*^ (▲, △), and *PIGCY*^*Tg*^*Y99C*^*Tg*^ (◇, ◆) mice at different ages; open symbols–individual animals; filled symbols with error bars–averaged (mean ± SD) for each age; data fit assuming exponential decay. *D*, The ONL thickness in the wild-type (○), *Y99C*^*Tg*^ (△), and *PIGCY*^*Tg*^*Y99C*^*Tg*^ (◇) mice aged 6 months; the *p* values are from ANOVA/Scheffe *post hoc*.

The age-related progression of photoreceptor loss in *Y99C*^*Tg*^ quantified *in vivo* using retinal OCT imaging (Fig. 4*C*) became remarkably slower in *PIGCY*^*Tg*^*Y99C*^*Tg*^ littermates. As *Y99C*^*Tg*^ retinas progressively lost more than half of the photoreceptor nuclei within 4 months, the photoreceptor nuclei mass in *PIGCY*^*Tg*^*Y99C*^*Tg*^ was reduced by less than 13% (Fig. 4*C*). After 6 months, the continued degeneration reduced *Y99C*^*Tg*^ rod nuclei layer thickness threefold, to 19 ± 5 μm (mean ± SD, n = 16), from 63 ± 2 μm in wild type (n = 18; ANOVA/Scheffe *post hoc p* < 0.0001). At the same age, in *PIGCY*^*Tg*^*Y99C*^*Tg*^ littermates, the outer nuclear layer thickness remained 84% normal, 53 ± 4 μm (*p* < 0.0001) (Fig. 4*D*).

### PIGCY^Tg^Y99C^Tg^ transgenic rods remain functional

Consistent with their dramatically improved survival, rod function in *PIGCY*^*Tg*^*Y99C*^*Tg*^ mouse retinas assessed by dark-adapted electroretinography (ERG) 32, 33 between 7 and 8 months of age was much better preserved than in *Y99C*^*Tg*^ (Fig. 5). Rod-specific photoresponse was tested with dim flashes isomerizing ∼17 rhodopsin molecules (R⁎) per rod (Fig. 5*A*). At this flash strength, less light-sensitive cones virtually do not contribute to the ERG waveform 32, 33, whereas rods generate well-defined scotopic “b-wave”—positive voltage deflection produced by the inner retina neurons receiving input from hyperpolarizing rods 32, 33. A bright (∼0.6 10^6^ R⁎/rod) flash (Fig. 5*B*), in addition to the b-wave, also generates a sharp “a-wave,” negative voltage deflection directly produced by reducing circulating current in photoreceptors 32, 33. Contribution to the a-wave from cones, which present a much smaller population of photoreceptors in mice, is still rather minor compared with much more abundant rods, whose response strongly dominates the bright-flash ERG waveform, such that the a-wave amplitude in dark-adapted mice also mostly reflects a rod response 32, 33.

**Figure 5:**
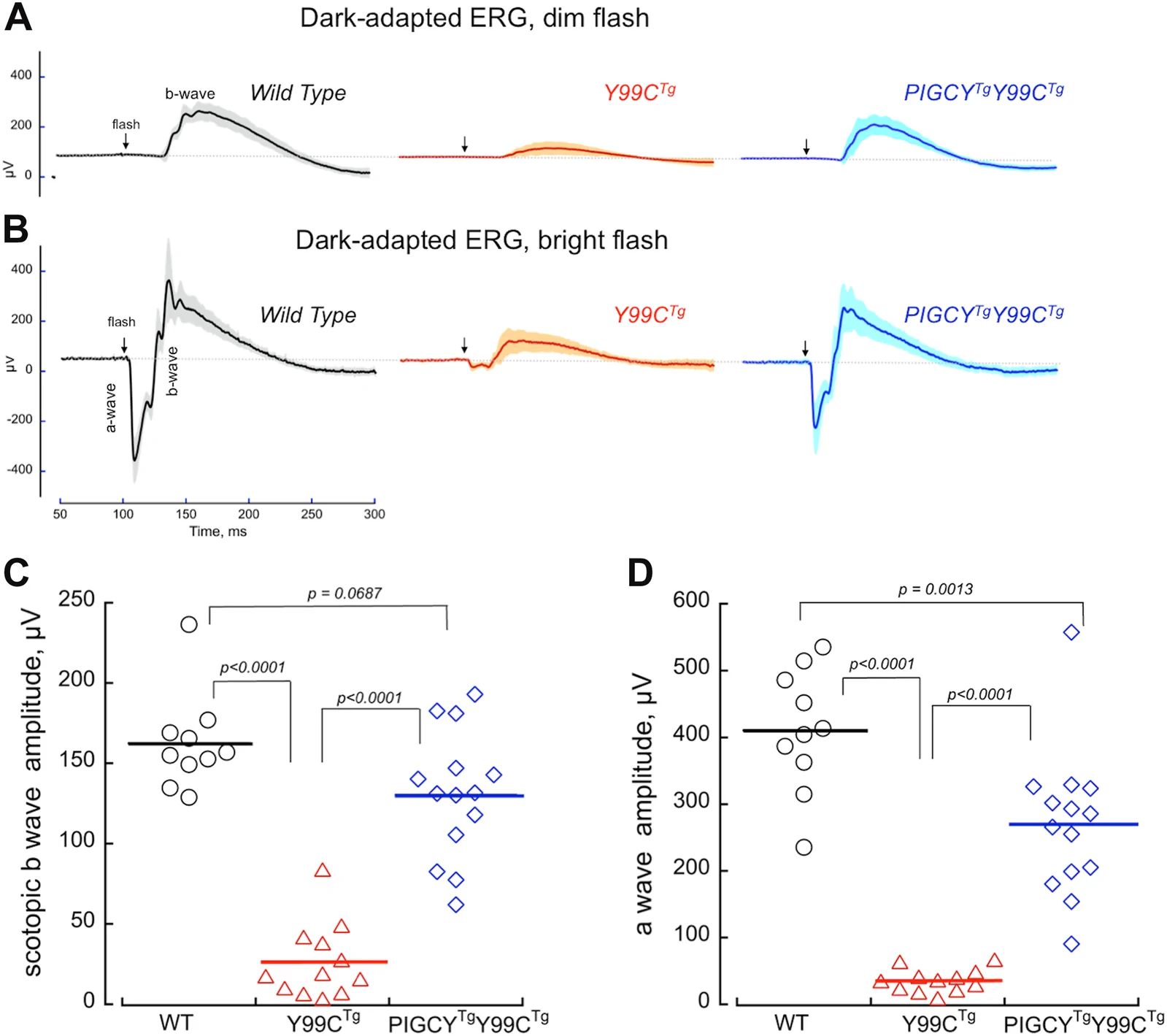
***PIGCY*^*Tg*^*Y99C*^*Tg*^ rods remain functional.***A* and *B*, dark-adapted ERG responses from 10 wild type (*black trace*), 12 *Y99C*^*Tg*^ (*red*), and 14 *PIGCY*^*Tg*^*Y99C*^*Tg*^ (*blue*) mice aged 7 to 8 months; solid traces–averaged responses, shaded areas–±SD. *A*, Rod ERG, 1-ms (*arrow*) 505 nm dim flash (17 R⁎/rod). *B*, rod-cone ERG, 1-ms 505 nm bright flash (∼0.6 10^6^ R⁎/rod). *C*, scotopic (17 R⁎/rod) rod ERG b-wave amplitudes in wild type (○), *Y99C*^*Tg*^ (△), and *PIGCY*^*Tg*^*Y99C*^*Tg*^ (◇) (mean ± SD): 162 ± 30 μV, 27 ± 23 μV, and 130 ± 39 μV, respectively. *D*, the a-wave (predominantly rod 32, 33) amplitudes to the ∼0.6 10^6^ R⁎/rod bright flash in wild type (○), *Y99C*^*Tg*^ (△), and *PIGCY*^*Tg*^*Y99C*^*Tg*^ (◇) (mean ± SD): 411 ± 92 μV, 36 ± 18 μV, and 269 ± 110 μV, respectively. The *p* values are from ANOVA/Scheffe *post hoc.*

Both dim-flash and bright-flash ERG waveforms were heavily suppressed in aged *Y99C*^*Tg*^ mice due to massive rod loss but remained well defined in *PIGCY*^*Tg*^*Y99C*^*Tg*^ littermates (Fig. 5, *A* and *B*). The scotopic b-wave amplitude was diminished sixfold in *Y99C*^*Tg*^ compared with the wild type (ANOVA/Scheffe *p* < 0.0001) but remained at approximately 80% of the normal amplitude in their *PIGCY*^*Tg*^*Y99C*^*Tg*^ littermates (*p* ∼ 0.069) (Fig. 5*C*). The a-wave to a bright flash was suppressed more than tenfold in *Y99C*^*Tg*^, down to 36 μV, from 411 μV in wild type (*p* < 0.0001), but retained 65% normal amplitude in *PIGCY*^*Tg*^*Y99C*^*Tg*^ (269 μV, *p* = 0.0013) (Fig. 5*D*).

## Discussion

PIGCY has been demonstrated to rescue degenerating rods (16) in a mouse model harboring R838S RetGC1 34, 35, one of many RetGC1 mutants causing dominant *GUCY2D* cone–rod dystrophy in humans 36, 37. Although the causal mutations arise from two different genes, *GUCY2D* and *GUCA1A* retinopathies share a common underlying mechanism: deregulation of the negative Ca^2+^ feedback on RetGC activity fails to suppress cGMP production at normal Ca^2+^ concentrations in dark-adapted photoreceptors, leading to abnormal cGMP accumulation in the outer segment and excessive influx of Ca^2+^ *via* CNG channels 11, 12, 34, 35. The only difference is that the abnormal Ca^2+^ sensitivity is imparted to the RetGC1:GCAP1 complex by the mutant cyclase itself in *GUCY2D* retinopathy (36) or its mutant calcium-sensor protein in *GUCA1A* retinopathy (Fig. 1 and references 7, 8, 9, 10, 21, 22, 23, 24). PIGCY reduces cGMP synthetic activity of the complex by substituting GCAP in the complex in both cases: in *GUCY2D* retinopathy – by uncoupling the mutant RetGC1 from the normal GCAPs (35), and in *GUCA1A* retinopathy – by uncoupling the normal RetGC1 from the mutant GCAP1 (Fig. 2). Despite the two-fold increase of IC_50_ in suppressing Y99C GCAP1-dependent stimulation of the cyclase *in vitro* (Fig. 2), PIGCY effectively inhibits cGMP synthesis in *Y99C*^*Tg*^rods (Fig. 3*B*), just as it does in wild-type retinas (15), and thus rescues *PIGCY*^*Tg*^*Y99C*^*Tg*^ rods (Fig. 4) like rescuing *PIGCY*^*Tg*^*R838SRetGC1*^*Tg*^ rods (16). This versatility supports the prospective use of PIGCY as a therapeutic agent for potential gene therapy for dominant retinopathies originating from the two genes.

Lowered Ca^2+^ sensitivity in mouse models harboring disease-causing RetGC1 and GCAP1 mutants elevates the cGMP-gated current and influx of Ca^2+^ 12, 35 sufficiently high to set in motion rod apoptosis. PIGCY-mediated inhibition decreases the deregulated RetGC activity to below normal (Fig. 3*B* and reference (15)), potentially depriving rescued photoreceptors of the phototransduction messenger and making them virtually dysfunctional. Such dysfunction could conceivably make the rescued photoreceptors resemble those in recessive *GUCY2D* Leber’s congenital amaurosis, blindness at birth in which photoreceptors in the retina are alive but lack sufficient cGMP production and lose photoresponse 38, 39, 40. However, because RetGC activity does not normally rate-limit the kinetics of rod photoresponse 32, 41, 42, even significant but not complete inhibition of RetGC activity by PIGCY does not incapacitate *PIGCY*^*Tg*^*Y99C*^*Tg*^ transgenic rods (Fig. 3*B*). Although the rate of cGMP synthesis in *PIGCY*^*Tg*^*Y99C*^*Tg*^ is substantially, nearly three-fold in low Ca^2+^ and nearly twofold at the “dark” Ca^2+^ levels, reduced (Fig. 3*B*), this reduction does not critically impair rod function (Fig. 5). Both scotopic b-wave and the a-wave to a bright flash are only partially diminished compared with the wild type. Hence, most of the rescued *PIGCY*^*Tg*^*Y99C*^*Tg*^ rods in the aged mice generate photoresponse, in stark contrast to *Y99C*^*Tg*^ littermates suffering massive rod degeneration. Single-cell suction electrode recordings in *PIGCY*^*Tg*^ and *PIGCY*^*Tg*^*R838S RetGC1*^*Tg*^ retinas (16) also reveal only minor changes in the parameters of rod photoresponse. Although further suppression of RetGC activity could potentially hinder the light sensitivity of photoreceptors, the threshold for PIGCY causing critical photoreceptor dysfunction is not reached in *PIGCY*^*Tg*^ rods and remains yet to be determined.

Considerations should also be given to the possibility of using PIGCY for treating diseases caused by insufficient hydrolysis of cGMP in the dark, rather than its excessive synthesis. The reduced catalytic activity of PDE6 could conceivably be compensated using the reduction of cGMP synthesis. PIGCY does not selectively uncouple only the RetGC1:GCAP1 complex in which one of the components presents a product of a mutant *GUCY2D* or *GUCA1A* alleles. PIGCY equally well inhibits normal RetGC:GCAP complexes 15, 16. The reduction of cGMP synthesis in rods harboring R838S RetGC1 or Y99C GCAP1 results from nonselective inhibition of both the normal and the mutant RetGC:GCAP complexes, because in neither case PIGCY reverses the reduced Ca^2+^ sensitivity of cGMP synthesis (Fig. 3*A*, and reference (16)). Therefore, the reduction of cGMP synthesis by the endogenous RetGC:GCAP complexes in photoreceptors with deficient cGMP hydrolysis appears plausible.

However, PIGCY’s ability to counteract disease-causing mutations may vary depending on the specific biochemical properties of the different GCAP1—and, possibly, RetGC1—mutants. PIGCY was engineered by introducing mutations in the GCAP1 backbone to increase its affinity for RetGC while disabling cyclase activation (15). Accordingly, PIGCY effectively uncouples the RetGC1:GCAP1 complexes containing GCAP1 mutants linked to *GUCA1A* retinopathy—E155G and the more frequent variant Y99C. Although the relatively modest increase in PIGCY IC_50_ for Y99C GCAP1 (Fig. 2) does not prevent rescue of *PIGCY*^*Tg*^*Y99C*^*Tg*^ rods (Figs. 4 and 5), the much higher IC_50_ required for counteracting G86R and L176F GCAP1 (Fig. 2) could pose a challenge for potential gene therapy in those cases. These two *GUCA1A* mutants not only display reduced Ca^2+^ sensitivity but also markedly increased affinity for RetGC1 23, 24, 26. Both substitutions were also used to engineer PIGCY itself (15), therefore, stronger competition of G86R or L176F GCAP1 with PIGCY (Fig. 2) is not unexpected. However, this difference illustrates that the therapeutic potential of PIGCY for any dominant GCAP1 mutant associated with retinopathy 43, 44, 45, 46 needs to be biochemically evaluated *in vitro*. If the affinity of any GCAP1 mutant for RetGC1 and *vice versa* markedly elevates PIGCY IC_50_, then other strategies might be better considered, such as using recombinant PDE5 targeted to the photoreceptor outer segment (13) to reduce the abnormally elevated cGMP levels. Also, although changes in Ca^2+^ sensitivity of the RetGC:GCAP complex caused by mutations in GCAP1 can be reliably detected *in vitro* using recombinant RetGC1 expressed in HEK cells (Fig. 1 and references 23, 36, 47), the overall Ca^2+^ sensitivity imparted by GCAPs to native RetGC in rod outer segment membranes is generally higher than in reconstituted HEK293 membranes (Fig. 3*A* and references 11, 12, 20, 21). Therefore, the extent to which different mutations shift Ca^2+^ sensitivity of cGMP synthesis in transgenic mouse retinas outside the physiological free Ca^2+^ range (27) more adequately reflects the deregulation of negative Ca^2+^ feedback causing *GUCA1A* retinopathy.

## Experimental procedures

### Animals

All experiments involving animals were conducted in accordance with the Public Health Service guidelines and approved by the Drexel University Institutional Animal Care and Use Committee. The wild-type C57BL/6J mouse strain originated from JAX Research/Jackson’s Laboratory. *Y99C*^*Tg*^ (line 52H) and *PIGCY*^*Tg*^ mice were produced as described previously 11, 15 and made congenic to the C57BL/6J background prior to conducting the experiments. Mice were housed using a standard 12 h/12 h light (ca.15-20 lux at the cage)/dark cycle and fed with the same diet (DietLab 5053).

### RetGC1 expression and guanylyl cyclase activity assay in HEK293 membranes

Recombinant human RetGC1 was expressed in HEK293 cells transfected with RetGC1 cDNA in a modified pRCCMV vector containing the cytomegalovirus promoter (originated from Invitrogen), and the crude membrane fractions containing RetGC1 were isolated using centrifugation as previously described (47) with modifications described in detail (48). Myristoylated recombinant GCAP1 and its mutants were expressed in *E. coli* and purified as previously described (29), with modifications described in detail (48). Recombinant myristoylated PIGCY was expressed in *E. coli* and purified as previously described in detail (15). RetGC activity in HEK293 membranes was assayed *in vitro* using [α–^32^P]GTP (Revvity) as a substrate, and the produced [^32^P]cGMP was analyzed by thin-layer chromatography on polyethyleneimine cellulose plates and scintillation counting of the radioactivity as previously described in detail (29).

### Guanylyl cyclase activity in the retina

The retinas for the RetGC activity measurements were excised under infrared illumination using a dissecting microscope fitted with infrared goggles from euthanized dark-adapted overnight, 1-month-old mice. The retinas were frozen in liquid N_2_ and stored at −70 °C prior to their use in the guanylyl cyclase activity assays, which were also conducted under infrared illumination as previously described in detail 15, 29. Briefly, the assay contained in 25 μl 30 mM MOPS-KOH (pH 7.2), 60 mM KCl, 4 mM NaCl, 1 mM DTT, 0.3 mM ATP, 4 mM cGMP, 1 mM GTP, 10 mM creatine phosphate, 0.5 unit of creatine phosphokinase (Sigma-Aldrich), ca. 1 μCi of [α-^32^P]GTP (Revvity), 0.1 μCi of [8-^3^H]cGMP (PerkinElmer), phosphodiesterase inhibitors zaprinast and dipyridamole, and 2 mM Ca^2+^/EGTA buffer 49, 50 with MgCl_2_ added to maintain variable [Ca^2+^]_free_ at physiological 0.9 mM free Mg^2+^ (51). The assay mixture was incubated for 12 min at 30 °C, and the reaction was stopped by heat-inactivation at 95 °C for 2 min. Data fitting was performed using Synergy Kaleidagraph 4 software.

RetGC1 presence in the retina was tested as described (15) by immunoblotting utilizing anti-RetGC1 antibody (52) and anti-peripherin-2 antibody (provided by Dr Andrew Goldberg, Oakland University).

### Optical coherence tomography (OCT) *in vivo*

Mice were anesthetized using intraperitoneal injection of ∼20 mg/kg Ketamine and ∼8 mg/kg Xylazine (Dechra). The pupils were dilated by applying 1% Tropicamide and 2.5% Phenylephrine ophthalmic eye drops ∼5 to 10 min before the scan. The A- and then B-scans of the retinas were conducted using an iiScience OCT camera calibrated by the manufacturer at 2.47 μm/pixel resolution axial scale and 3.5 μm/pixel lateral scale and averaged from ∼200 frames using ImageJ/Fiji software (National Institutes of Health). For consistency of the measurements, the thickness of ONL was measured in all mice between the outer plexiform and the external limiting membrane reflective layers 53, 54 in B-scans across the same part of the retina, ca. 400 to 700 μm below the optic nerve.

### Retinal post-mortem morphology

Mice were anesthetized with a lethal dose of Ketamine/Xylazine injection, perfused through the heart with phosphate-buffered saline (PBS) and then with 2.5% glutaraldehyde/3% paraformaldehyde (Electron Microscopy Sciences) in PBS. The eyes were surgically removed and fixed overnight in 2.5% glutaraldehyde/3% formaldehyde in PBS at room temperature. The fixed eyes were washed in PBS, soaked in PBS overnight, processed for paraffin embedding, sectioned at 5-μm and stained with hematoxylin/eosin (AML Laboratories). The retinal sections were photographed using an Olympus BX21 microscope fitted with an Olympus MagnaFire camera.

### Electroretinography (ERG)

Mice were dark-adapted overnight, their pupils were dilated by applying 1% Tropicamide and 2.5% Phenylephrine ophthalmic eye drops under dim red safelight illumination, and the mice were dark-adapted for another 10 min. Full-field ERG in mice anesthetized by inhalation of 1.7 to 1.9% Isoflurane (VEDCO)/air mix delivered by a Kent Scientific SomnoSuite setup at 50 ml/min was performed in the dark as previously described 15, 16 using a Phoenix Research Laboratories Ganzfeld ERG2 instrument. Light flashes (1-ms, 505 nm) were delivered through the infrared camera-guided corneal electrode/LED light source of the instrument.

## Statistics

Where applicable, statistical significance of the differences was tested by ANOVA/Scheffe *post hoc* (alpha 0.01) or unpaired Student’s *t* test using a Synergy Kaleidagraph software. The pertinent statistical differences are shown in *Results*.

## Data availability

The data referred to in this manuscript are contained within the manuscript. Unprocessed data can be available upon reasonable request from the corresponding author (ad4293@drexel.edu).

## Supporting information

This article contains supporting information.

## Conflict of interest

The authors declare that they have no conflicts of interest with the contents of this article.
