# Supplementary material for: Protein inhibitor of retinal membrane guanylyl cyclase rescues photoreceptors from degeneration induced by mutated guanylyl cyclase activating protein
Source: J Biol Chem. 2026 Oct 14;302(12):113636. doi: 10.1016/j.jbc.2026.113636 (PMC13769578; doi:10.1016/j.jbc.2026.113636)
Supplement: Supplementary file 1 [file mmc1.docx]

**SUPPLEMENT**

**Protein inhibitor of retinal membrane guanylyl cyclase rescues photoreceptors from degeneration induced by mutated guanylyl cyclase activating protein**

Lyuqi Tan^1,2^, Igor V. Peshenko^1^, Elena V. Olshevskaya^1^, and Alexander M. Dizhoor^1,3*#^

*^1^Pennsylvania College of Optometry, Drexel University; ^2^Graduate School in Biomedical Sciences and Professional Studies, College of Medicine, Drexel University; ^3^Department of Neurobiology and Anatomy, College of Medicine, Drexel University, United States*

**Supplemental Figure S1. Electrophoretic mobility of Y99C GCAP1 and PIGCY used for transgenic expression in mouse rods**. Purified recombinant proteins were separated on a 4–20% Tris-Glycine gradient SDS-PAGE gel and visualized by Coomassie blue staining. Left to right: molecular mass markers, 1 µg mouse GCAP1, 1 µg Y99C GCAP1, 1 µg PIGCY, and a mixture of 0.5 µg Y99C + 0.5 µg PIGCY. Note that whereas Y99C GCAP (reference **11**) and PIGCY (reference **15**) can be distinguished from mouse GCAP1 by their slightly slower electrophoretic mobility, Y99C GCAP and PIGCY have virtually identical mobility and cannot be independently quantified when present together.
